# Review of evidence that foxes and cats cause extinctions of Australia's endemic mammals

**DOI:** 10.1093/biosci/biaf046

**Published:** 2025-04-10

**Authors:** Arian D Wallach, Erick J Lundgren

**Affiliations:** School of Biology and Environmental Science, Faculty of Science, Queensland University of Technology, Brisbane, Queensland, Australia; Center for Open Science and Synthesis in Ecology and Evolution, Department of Biology, University of Alberta, Edmonton, Alberta, Canada

## Abstract

Over half of Australia's threatened and extinct endemic mammal species have been attributed to introduced red foxes (Vulpes vulpes) and cats (Felis silvestris catus). But this claim has so far been based on expert opinion. We conducted a timeline analysis, systematic review, and meta-analysis to assess whether the attribution of decline and extinction to these predators is based on evidence. Records for 43.6% and 19.6% of populations did not confirm that extinctions occurred after fox and cat arrival, respectively. Most threatened species have been attributed to these predators without supportive population studies with data (76.1% of species attributed to foxes, and 79.7% to cats). The meta-analysis showed a negative correlation between threatened mammal and fox abundance for spatial but not for temporal correlations, and we found no evidence for a correlation with cats. We conclude that the hypothesis that foxes and cats cause extinctions has come to be accepted with little evidence.

The conservation community has galvanized around the claim that introduced species are one of the main global causes of extinction (Chew 2015). Introduced mammalian predators are considered the worst offenders, blamed for over half of the world's mammal, bird, and reptile extinctions (Doherty et al. 2016). Even the staunchest critics of invasion biology, who have long argued that there is an entrenched bias against introduced species, concede that introduced predators might indeed “represent a major extinction threat” (Davis et al. 2011). The loss of Australia's endemic mammals is one of the world's prominent cases. Red foxes (*Vulpes vulpes*) and wild cats (*Felis silvestris catus*) are attributed with causing the highest number of extinctions in Australia since the late Pleistocene (Johnson 2006) and have been included among 100 of the World's Worst Invasive Alien Species, a blacklist of introduced species compiled by the International Union for the Conservation of Nature (IUCN; Lowe et al. 2000).

Blaming foxes, cats, and other “introduced enemies” for the extinction of Australia's “gentle and highly specialized creatures” (Troughton 1938) dates back at least a century (Hoy 1923, Le Souef et al. 1926, Troughton 1938, Rolls 1969). Today, it is accepted as a self-evident truism. Saunders and colleagues (2010) concluded that there is “overwhelming evidence that foxes have negative impacts on a very broad range of native Australian vertebrates,” and Legge and colleagues (2017) claimed that the “evidence of marked detrimental impacts of feral cats is irrefutable.” It is also widely agreed that foxes and cats cause extinctions above and beyond other potential causes. Doherty and colleagues (2017) claimed that cats are a “primary” driver of mammal extinctions globally, Woinarski and colleagues (2019) similarly stated that foxes and cats are “primary” drivers of extinction in Australia, and Medina and colleagues (2011) stated that cats are the “principal threat” to vertebrates on islands worldwide.

We agree that it is a reasonable hypothesis that predators can contribute to the decline of their prey and that, in specific cases, this might contribute to the process of extinction. But it is also reasonable to expect that the evidence would match what has come to be regarded as an “overwhelming scientific consensus” (Loss et al. 2018). To the best of our knowledge, there are seven articles synthesizing the effects of foxes and cats on Australia's endemic mammals. All of these are based on expert opinion (table 1). Some can appear empirical by converting qualitative rankings from expert assessments (e.g., Red List) into quantitative data, which then forms the basis of analysis, even if there is little or no evidence to support the assessment. For example, Doherty and colleagues (2016) described foxes and cats as “important driver[s] of Australian mammal species extinctions,” and cats as “major agents of extinction” globally, even though their analysis of 29 of 38 species (76%) attributed to foxes and 46 of 54 species (85%) attributed to cats “had no evidence” to support this assessment (summarised from Doherty et al. 2016). Similar reliance on expert opinion to attribute the decline of hundreds of amphibian species to the chytridiomycosis disease was criticized by Lambert and colleagues (2020). Reliance on expert opinion is not new. Nearly three decades ago, Wilcove and colleagues (1998) pointed out that “assessments of the threats to individual species are often based on the subjective opinions of knowledgeable individuals, rather than experimental evidence.” Expert opinion can be useful, but it is prone to bias, groupthink, and overconfidence (Burgman 2004, Burgman et al. 2011), and it should not be confused with evidence.

**Table 1.** All syntheses of the impacts of red foxes and cats on groups of native prey are based on expert opinion.

Study	Taxa declining	Region	Attributed predator	Decline attributed to predator	Study resources	Expert opinion	IF	Cites
Bellard and colleagues (2016)	Terrestrial vertebrates	Global	Cats	Third most common introduced species threat	Red List threat categories	“We compiled the external threats for each species from the Red List”	5.53	>200
Doherty and colleagues (2016)	Terrestrial vertebrates	Global	Cats	63 extinctions, 430 endangerments	Qualitative author assessment of Red List and literature review	“Given the difficulties in attributing causation in species declines and extinctions, most inferences regarding the impact of invasive predators were based on observational evidence, rather than experimental data. For this reason, we… coded the degree of predator impacts”	12.78	>1,100
		Australia	Foxes	9–11 extinctions, 48 endangerments				
Medina and colleagues (2011)	Terrestrial vertebrates	Global	Cats	14% (terrestrial) extinctions, 8% endangerments	Qualitative author assessment of literature review	“a categorical series of qualitative weights assigned to each case”	13.21	>600
Radford and colleagues (2018)	Terrestrial mammals	Australia	Foxes and cats	36% “extremely” or “highly” susceptible	Qualitative author assessment of Red List and literature review; and expert opinion	“predator response information was derived from… published and unpublished (e.g., expert opinion, local knowledge and government, non-government and community groups associated with translocation and predator control programs) sources… [For predator susceptibility] we convened a workshop”	2.18	70
Szabo and colleagues (2012)	Birds	Global	Introduced species (mainly predators)	58% of species	Red List threat categories	“The threats believed to have driven each taxon extinct were coded using the IUCN Red List”	3.75	>180
Woinarski and colleagues (2019)	Terrestrial vertebrates	Australia	Cats	97 extinctions and endangerments	Qualitative expert assessment including Woinarski and colleagues (2014) and literature review	“we reviewed all available information… to derive a draft table of estimated impacts… of threat factors…. Ratings [were] revised… in light of… expert responses”	7.5	>140
	Terrestrial vertebrates	Australia	Fox	58 extinctions and endangerments				
Woinarski and colleagues (2015)	Terrestrial vertebrates	Australia	Foxes and cats	33 extinctions and endangerments	Qualitative literature and author assessments; Red List; legislation assessments; and the author's opinion	“[We used] Red List accounts, listing advices… and reviews… to make an assessment… For every extinct species, at least three of the co-authors… assigned their assessments”	12.78	900

*Note:* The articles assessed the effects of introduced predators (“attributed predator”) on threatened taxa (“taxa declining”) at a local or global scale (“region”) and concluded the scale of decline these predators are responsible for (“decline attributed”). All the articles used qualitative sources (“study resources”), such as the IUCN Red List (e.g., Hayward et al. 2015), and other opinion-based metrices (see “direct quote”) as the basis for their conclusions (“basis”). Journal impact factor (“IF”), a measure of citation rate of the journal, is regarded as a measure of journal prestige, and citation amount (“cites,” Google Scholar, February 2024) indicates article influence.

We took a fresh and systematic look at the accumulated primary evidence associating foxes and cats to threatened and extinct Australian mammals. We conducted a timeline analysis, an exhaustive systematic review, and a meta-analysis. Our aim was to make transparent the evidence available to support a foundational claim of conservation biology.

## Do we know whether extinctions occurred after predator arrival?

Foxes and cats have been attributed with 57% of Australian mammal threatened status (46 species attributed to foxes, 64 to cats) and 68% of extinctions (11 to foxes, 17 to cats). As a starting point, we must be reasonably certain that the extinctions (and local extirpations) attributed to foxes and cats occurred after they were established in Australia. We found the locations and years of the last confirmed records for 164 populations of 52 species (25 extinct species; 19 threatened species, of which 9 are extinct on the mainland; and 8 least concern species with local extirpation records) and compared them with timeline estimates of the arrival and spread produced by Fairfax (2019) for foxes and by Abbott (2008) for cats (supplemental table S1).

**Figure 1.**
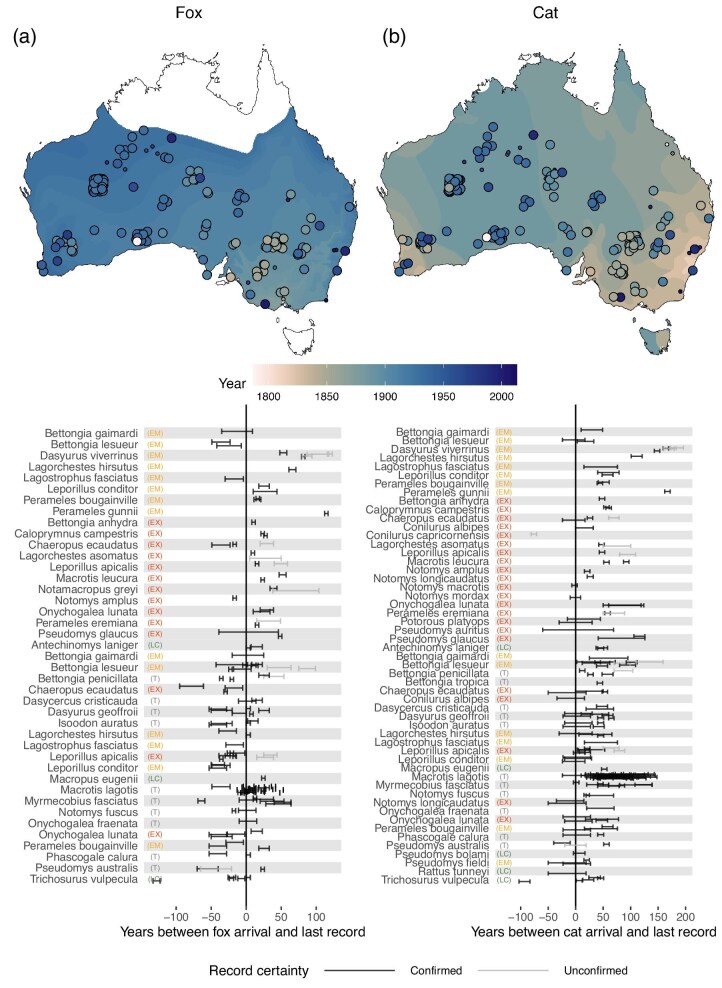
Last records of extirpated populations per species relative to earliest local estimates of foxes and cats, by location (a) and year (b). In panel (a), the contour and point colors show the record year; large points show locations of confirmed records, small points of unconfirmed records (excluded from analyses); and the points locate the center of record regions. In panel (b), the error ranges account for documented uncertainty in the time and place of extirpation and arrival records. Species are included if foxes and cats were listed as a threat or cause of extirpation or extinction in the IUCN Red List (IUCN 2024) or the EPBC (EPBC 2013, 2015). The earliest local records of foxes were sourced from Fairfax (2019) and of cats from Abbott (2008), from which the maps (a) were adapted. The records are detailed in supplemental table S1. Polygons for imprecise extirpation records are shown for each species individually at https://ejlundgren.github.io/introduced_predators_evidence/. See supplemental figure S1 for assessment of spatial clustering of records and supplemental figure S2 for summaries for EX, T, EM, and LC species. Abbreviations: EM, threatened and extirpated on the mainland; EX, extinct; LC, least concern; T, threatened (including near threatened, vulnerable, endangered, and critically endangered).

The timeline comparison showed that the available evidence for 31% of the populations does not confirm that their extirpations occurred after the predators’ arrival (figure 1a). Of the extirpated populations attributed to foxes, 43.6% could not be confirmed to have been lost after the foxes’ arrival: 35.4% were last recorded before the earliest estimate of the foxes’ arrival (populations per species, mean [*M*] = 56.9, standard deviation [SD] = 26.6%, range 14.3%–100%), and an additional 8.2% of the records were uncertain whether the extirpation occurred before or after (*M* = 46.7, SD = 34.9%, range = 1.9%–100%). Of the extirpated populations attributed to cats, 19.6% could not be confirmed to have been lost after the cats’ arrival: 1.2% were last recorded before the earliest estimate of cat arrival (*M* = 35, SD = 21.2%, range = 20%–50%), and an additional 18.4% were uncertain (*M* = 54.2, SD = 34.5%, range = 1.9%–100%; figure 1b). We found no evidence that the extirpation records that predate the predators’ arrival estimates were uniquely clustered (supplemental figure S1).

The confirmed records also suggest that eight species (*Bettongia lesueur, Dasyurus viverrinus, Lagorchestes hirsutus, Macrotis lagotis, Myrmecobius fasciatus, Onychogalea lunata, Perameles gunnii*, and *Pseudomys glaucus*) had populations that co-occurred with these predators for over a century before extirpation. One population of *P. gunnii* co-occurred with foxes for over a century (3.1% of the species with records, 0.7% of the populations); six populations of five species co-occurred with cats for over a century (10.4%, 3.7%), and an additional 36 populations of five species had records that spanned before and after a century with cats (10.4%, 22.1%; figure 1b).

## Have threatened mammals been attributed on the basis of ecological research?

**Figure 2.**
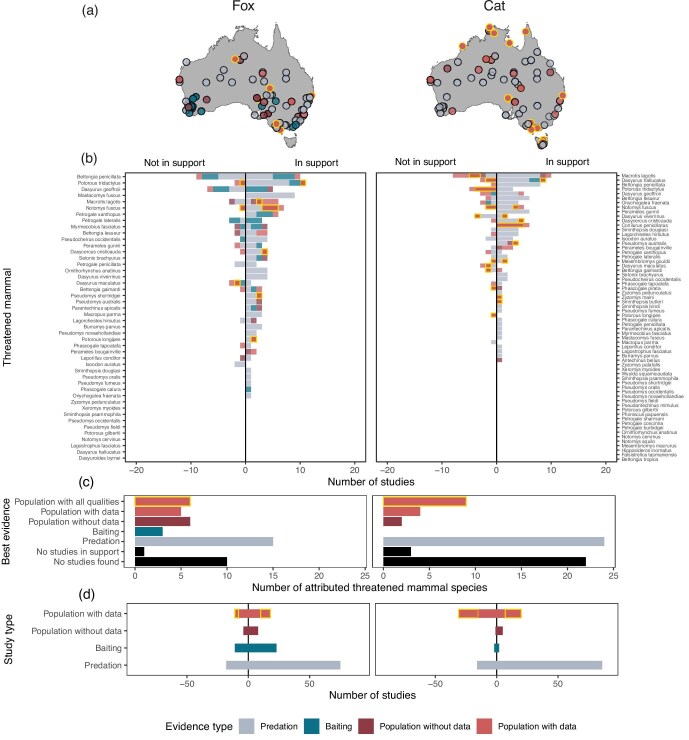
Systematic review of evidence for an association between attributed threatened mammals and foxes and cats. Positive studies are in support of the hypothesis that foxes and cats are a cause of the decline or rarity of threatened mammals, studies on the negative scale are not in support. Distribution of studies (a); number and type of studies found for each threatened mammal (b); number of species in each supportive evidence category (c); number of studies in support and not in support for each evidence category (d). Panel (c) counts the number of threatened mammals for whom no studies were found (black) or all studies found were not in support (black); for whom predation studies (gray), baiting studies (blue), and population studies without data (dark red) were the best supportive evidence found; and species for whom supportive population studies with data (red), and with all key qualities (data, sample size, control, confounders) in support were found (red with yellow border). See table 2 for study categories, and species summaries in supplemental table S2 or the summary website https://ejlundgren.github.io/introduced_predators_evidence/. Species were included if foxes or cats were listed as a threat or cause of decline or extinction by the IUCN Red List (IUCN 2024) or by the EPBC Threat Abatement Plans (EPBC 2013, 2015).

We conducted an exhaustive systematic review (supplemental figure S3) to find out whether the threatened status of mammals has been attributed to foxes and cats on the basis of evidence. We uncovered 163 articles reporting 331 unique studies (observations, reports, experiments, data sets) from across Australia (figure 2a, supplemental table S2). We grouped the studies into three main categories: predation, baiting, and population (table 2). The predation studies have no data on predator or prey populations. These studies can only confirm whether foxes and cats are a cause of individual mortality. The baiting studies have no data on predator populations. These studies associate poison baiting programs with threatened mammal abundance but do not identify whether the target predator population declined. The hypothesis cannot be evidenced by predation and baiting studies because both lack data on predator or prey populations. The population studies provide information on both predator and prey populations. Within this category, we distinguished between three types of evidence: descriptive population studies (population studies with no data); those that report predator and prey data, such as abundance or occupancy (population studies with data); and those that have predator and prey data, a sample size greater than one, a control population or spatial variation, and include at least one confounding variable (population studies with qualities). This last category identifies the strongest evidence we found for testing predator–prey interactions, because we did not find any studies with a full experimental design (e.g., the before–after control–impact design). We categorized all studies as being in support or not in support of the hypothesis, and we defined study type (table 2). We also separately synthesized the evidence by the Australian government’s Threat Abatement Plan threat ratings, which qualitatively defines foxes and cats from minor to extreme threat to each threatened mammal species (DEWHA 2013, DCCEEW 2015).

**Table 2.** Systematic review categorization of studies of interactions between threatened mammals and foxes and cats.

Evidence category	Study type	Study in support of the hypothesis	Study not in support of the hypothesis	Evidence strength
Predation	Diet	Threatened mammal identified in the predator's diet (includes scavenging)	Study reports that the threatened mammal was present but not identified in the predator's diet	Weakest form of evidence. Limited to individual mortality or group mortality rate. Lacks predator and prey population data.
	Hunted (reintroduced)	Identified as a predator of reintroduced threatened mammal	Study reports that the predator was present but did not hunt reintroduced individuals	
	Hunted	Identified as a predator of locally born threatened mammal	Study reports that the threatened mammal was present but was not found to be hunted by the predator	
	Preference	Threatened mammal was identified as preferred prey	Threatened mammal was not identified as preferred prey	
Baiting	Baiting (temporal)	Threatened mammal abundance consistently increased following poison-baiting	Threatened mammal abundance did not consistently increase following poison-baiting	Weak form of evidence. Replaces predator data with proxy (poison baiting) of unknown reliability.
	Baiting (spatial)	Threatened mammal abundance was higher at poison-baited sites compared to control	Threatened mammal abundance was not higher at poison-baited sites compared to control	
Population	Timing	Extirpation or decline followed predator establishment or increase	Extirpation or decline preceded predator establishment or increase	Reports on predator and prey populations. Stronger evidence come from studies that report predator and prey data (rather than observations), have a sample size greater than one site or population, include a control or spatial variation, and report on at least one confounding or alternative variable.
	Exclusion	Abundance of threatened mammal was higher where predator was excluded	Abundance of threatened mammal was not higher where predator was excluded	
	Temporal	Temporal correlation between threatened mammal and predator abundance was negative	Temporal correlation between threatened mammal and predator abundance was not negative	
	Spatial	Spatial correlation between threatened mammal and predator abundance was negative	Spatial correlation between threatened mammal and predator abundance was not negative	

The systematic review did not show population studies with data in support of the hypothesis for most of the threatened mammals. Of the threatened mammals attributed to foxes, no studies of any kind in support of the hypothesis were found for 23.9% of the species (no studies = 21.7% of species and only studies not in support = 2.2%), and only weak supportive studies lacking predator or prey data were found for 52.2% of the species (best support found was predation = 32.6%; baiting = 6.5%; and population studies without data = 13%). This leaves 11 species attributed to foxes (23.9%) with supportive studies reporting predator and prey data (population studies with data = 10.9% and population studies with qualities = 13%; figure 2b, 2c). Similarly, of the threatened mammals attributed to cats, no supportive studies were found for 39.1% (no studies = 34.4%; only not in support = 4.7%), and only weak supportive studies lacking predator and prey data were found for 40.6% of the species (only predation studies = 37.5%; population studies without data = 3.1%). This leaves 13 species attributed to cats (20.3%) with supportive studies reporting predator and prey data (population studies with data = 6.3%; with qualities = 14.1%; figure 2b, 2c).

The systematic review also revealed that a substantial proportion of the studies were not in support of the hypothesis. Nearly half (47.5%) of the population studies with qualities were not in support (*M* = 45.8% of the studies per species, SD = 44.1%; 27.3% of the fox studies, 55.2% of the cat studies; figure 2d). In addition, a third of the baiting studies (34.2%) did not show a positive association between poison baiting and threatened mammal abundance (*M* = 34.9%, SD = 42.3%; fox = 32.4%; cat = 50%; figure 2d); and 17.4% of predation studies reported that threatened mammals were not eaten, hunted, or preferred (*M* = 17.9%, SD = 32.7; fox = 19.4%; cat = 15.7%; figure 2d). The most common study types in support were that threatened mammals were found in the diet of foxes and that cats hunt reintroduced animals (supplemental figure S4).

A similar pattern emerges when focusing on the threatened mammals ranked by the Australian Department of Climate Change, Energy, the Environment, and Water (DCCEEW 2015) as being at the highest risk from foxes and cats (very high to extreme). Of the 18 species defined at these high-risk categories from foxes, 13 (72%) did not have supportive population studies with data, and of the 23 species defined at high risk from cats, 18 (78%) did not have supportive population studies with data (supplemental figure S5).

## Are threatened mammal populations negatively correlated with foxes and cats?

If foxes and cats are a cause of the low abundance of threatened mammals, we would expect to find a negative correlation between predator and threatened mammal abundance. From the systematic review database, we were able to source 45 predator–prey abundance data sets from 19 articles of 14 threatened mammal species for inclusion in a meta-analysis.

**Figure 3.**
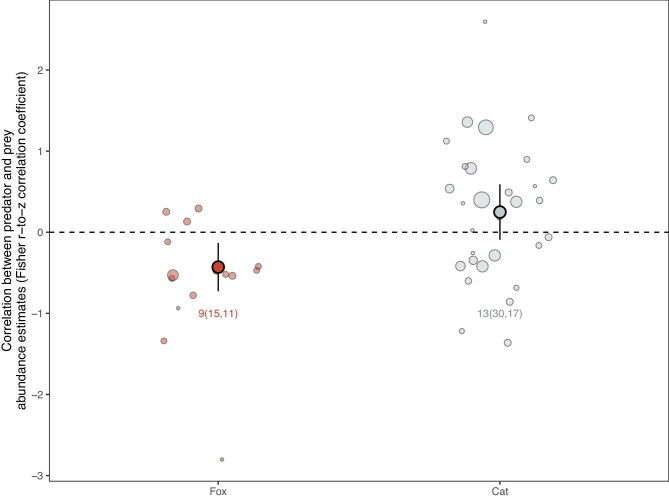
Meta-analysis of abundance correlations between threatened mammals and foxes and cats. Light shading, individual study effect sizes; circle size, proportional to data weight in the analyses; dark shading, model estimates of the overall effect of correlations across studies. The error bars represent 95% confidence intervals. The numbers represent the number of species and, in parentheses, the number of correlations followed by the number of articles. Sampling variance is determined by sample size and error.

Multilevel mixed effect meta-analytic models revealed evidence of a negative correlation with foxes (Fisher's *r*-to-*z* transformed correlation coefficient, *z* = .43, 95% confidence interval [CI]= –.73 to –.13, *p* = .009; figure 3). The correlation between foxes and prey was found in the spatial correlations (*z*  = –.57, CI = –.95 to –.19, *p* = .01) but not in the temporal ones (*z* = –.16, CI = –.81 to .49, *p* = .48; supplemental figure S6). Correlations were not found for cats when considering all data (*z* = .25, CI = –.09 to .59, *p* = .14) or when analyzing spatial and temporal correlations separately (spatial, *z* = .19, –.32 to 0.69, *p* = 0.42; temporal, *z* = .30, CI = –.21 to .82, *p* = .21; figure S6). These results were consistent when accounting for prey species identity or phylogenetic similarity (fox, *z* = –.43, *p* = .01; cat, *z* = .25, *p* = .16).

## Is the evidence sufficient?

We collated an exhaustive list of anecdotes, reports, data sets and analyses that link foxes and cats to Australia's threatened and extinct mammals. We synthesized these accounts in three ways: We asked whether there is evidence that extinctions occurred *after* predator arrival, whether the species’ threat status was attributed to these predators on the basis of population studies, and whether the predator and the threatened mammal’s abundance are negatively correlated. The strongest support for the hypothesis we found was a negative (spatial) correlation between foxes and the threatened mammal abundance (figure 3). However, we also found that over 40% of the reports place extinctions (and extirpations) before the foxes’ arrival estimates (figure 1) and that most threatened species (76%) have no population studies with data associating their decline to foxes (figure 2). There is even less evidence that cats contribute to extinctions: The reports place around a fifth of the extinctions before the cats’ arrival estimates and a similar number were lost after more than a century of co-occurrence (figure 1), for most threatened species (80%) no population studies with data were found associating their decline to cats (figure 2), and we found no evidence of a negative correlation between cat and threatened mammal abundance (figure 3).

What are we to conclude from the accumulated evidence? Before addressing this question, we ought to emphasize that the goal of our study was neither to prove (i.e., accept) nor disprove (i.e., reject) the hypothesis that foxes and cats cause extinctions, neither as a general rule nor for individual cases. Instead, the goal was to find out whether a “consensus” (Loss et al. 2018) has formed around a hypothesis because the evidence is “incontrovertible” (Losos 2019). For example, it is not possible to reject the hypothesis even in those cases where records place extinctions before predator arrival, because records do not necessarily reflect reality, but we can conclude that the claim that extinctions “followed the arrival and spread of these predators” (Johnson et al. 2007) oversteps the available evidence. By collating and synthesizing the primary evidence, we aimed to make it straightforward for those interested to draw their own conclusions around whether the evidence attributing extinctions and declines to introduced predators is persuasive (rather than relying on expert opinion; table 1). To further this purpose, we created an online summary of the evidence found for each species (accessible at https://ejlundgren.github.io/introduced_predators_evidence/). However, we also offer our own explanation for why, in our view (Lebowski 1998), the evidence as a whole and for each attributed threatened mammal is insufficient. For the purpose of this discussion, we will assume that we have been successful at identifying at least the vast majority of relevant studies.

The main reason we conclude that the evidence is insufficient is that the systematic review revealed that both predators have been attributed with threatened species decline without supportive population studies with data (figure 2). For example, foxes and cats are characterized as a very high threat to *Burramys parvus* by the DCCEEW (2015), on the basis of only possum remains in predator scats, and foxes are considered an extreme threat to *Petrogale lateralis* (DCCEEW 2015), on the basis of baiting studies that did not report data on foxes. Also notable is that in spite of the well-known tendency to publish positive results, the systematic review showed that half (51%) of the studies reporting predator and prey population data were not in support of the hypothesis. For example, cats are considered a high threat to *Potorous tridactylus* (DCCEEW 2015), even though the population studies did not support this rating. See species summaries at https://ejlundgren.github.io/introduced_predators_evidence/.

For most species whose threatened status is attributed to foxes and cats, this is at least plausible. But it is worth noting that there are some species for which this attribution not only lacks evidence but is also implausible. Foxes and cats might occasionally hunt challenging prey (e.g., McComb et al. 2018), but they are not known to regularly hunt animals that are arboreal (e.g., *Wyulda squamicaudata*), aquatic (e.g., *Ornithorhynchus anatinus*), flying (e.g., *Phoniscus papuensis*), or larger than themselves (e.g., the 3–8-kilogram cat is defined as a high risk to the 6–11 kilogram *Petrogale xanthopus*; Lanszki et al. 2007, Sunquist and Sunquist 2017, Faurby et al. 2020, Middleton et al. 2021), and we found no evidence that they do. See species summaries at https://ejlundgren.github.io/introduced_predators_evidence/.

This still leaves 20 species that have population studies with data in support of the hypothesis, some of which reported compelling analyses. In these cases, there can be legitimate disagreement about what constitutes sufficient evidence. The first and, in our view, strongest example is the evidence linking the decline of brush-tailed rabbit rats (*Conilurus penicillatus*) to cats. We identified six population studies, all in support, of which five met basic qualities for testing predator–prey interactions (the highest number of supportive studies that reported predator and prey data, had a sample size greater than 1, included a control or spatial variation, and considered confounding variables). We agree that it would be fair to conclude that there is reasonable evidence that cats are a suppressive force of rabbit rats in the Tiwi Islands. We do not agree that one can go further to claim that there is sufficient evidence that cats are the cause of the threatened status of rabbit rats, because the studies are not fully independent (the articles overlap in data, study sites, and authors). Two additional studies stand out. Abbott (2001) compiled detailed historic records of the last bilby (*Macrotis lagotis*) and the first fox observations and reported a positive correlation between them, supporting the proposition that the range of bilbies contracted as foxes spread. However, nearly a third of the records placed extinctions before fox arrival, and no confounding variables were considered. Finally, Moseby and colleagues (2020) recorded plains rat (*Pseudomys australis*) abundance along a transect running from inside to outside a predator-proof enclosure. Rats were regularly recorded inside the enclosure and abruptly became rare at the fence. Because the fence did not limit the rats’ movement, it seems evident that the presence of foxes and cats outside the fence was the culprit. However, from a single transect it is not possible to conclude that foxes and cats are the cause of a species’s threatened status (nor did Moseby et al. 2020 make this claim) particularly because it is not an unusual observation. Similar patterns have been reported when native predators where fenced out (e.g., Wilson et al. 1999, Ekerholm et al. 2004). It is also worth noting that one (of two) timeline records place the plains rats’ extirpation 40–70 years before the foxes’ arrival. (See species summaries at https://ejlundgren.github.io/introduced_predators_evidence/).

The negative correlation between fox and threatened mammal abundance and the lack of correlation with cats require careful consideration. It is fair to regard this analysis as evidence that foxes exert top-down pressure on these animals, but there are several important limitations to what can be concluded from this data. First, although the spatial correlation between foxes and threatened mammals was negative, the temporal correlation was not. Similarly (although in reverse), in a meta-analysis, Holt and colleagues (2008) found a temporal correlation but not a spatial correlation between predator and prey in the United Kingdom. Such inconsistencies weaken any conclusion that can be drawn from an analysis, unless a confounding variable can be clearly tested for. Second, the sample of species for which suitable data sets were available concentrated in the larger body mass range of attributed species (supplemental figure S7). This could, for example, hide negative correlations between cats and smaller prey. Finally, negative predator–prey associations are, of course, not unique to introduced predators (e.g., Holt et al. 2008, Katano et al. 2015). Suppressive effects of predators on their prey is the very basis of predator–prey dynamics, top-down control hypothesis, and trophic cascades (e.g., Ripple et al. 2016).

There are three additional lines of evidence that have been brought in support of the hypothesis that are important to consider. The first is that several analyses have shown that extinctions have tended to concentrate within the body mass range these predators hunt and in the arid zone where these predators can be more common (Burbidge and McKenzie 1989, Johnson 2006, Johnson and Isaac 2009, Legge et al. 2017). Whether extinctions have concentrated in a ‘critical weight range’ has been rejected by some studies (e.g., Cardillo and Bromham 2001). However, assuming it is valid, this is the most compelling line of evidence, in our view. It suggests that extinctions are not random and that they fit with what would be expected if foxes and cats did cause extinctions. Although there could be other reasons why extinctions concentrated in this body mass and in that region, to our knowledge, no competing hypotheses have been put forward. The second is a meta-analysis by Salo and colleagues (2007), in which they found a larger effect size between introduced predators and their prey than between native predators and their prey. The article title states that “alien predators are more dangerous than native predators.” This conclusion is premature, in our view, because the effect size in the introduced group was driven by four articles reporting Australian baiting studies, which lack predator data. Third, there are species that persist only on islands devoid of foxes and cats or that have been successfully introduced into fenced reserves that exclude the predators (Tulloch et al. 2022). Although it is compelling, this line of evidence has its own limitations: Predator-free islands and reserves differ in more ways than the presence of introduced predators (Cronk 1997), and similar increases in prey abundance have been observed where the excluded predators are native (e.g., Johnson et al. 2019, Theunissen 2019, Scoleri et al. 2023). Additionally, some have pointed out that predation by foxes and cats can cause reintroduction failures (Moseby et al. 2011). Although this observation is relevant to the practice of reintroductions, it is not relevant to the question of why extinctions occur. The fate of animals translocated or released from captivity is not a proxy for population dynamics (Armstrong and Seddon 2008), and reintroduction programs are foiled by native predators as well (e.g., Grey-Ross et al. 2009, Wimberger et al. 2009, Bennett et al. 2012).

Equally important to consider are two weaknesses in the argument that introduced predators, unlike native predators, drive the extinction of their prey. First, there is no evidence for a mechanism. For decades, it was accepted that animals succumb to introduced predators because they are naive to them or in some other way maladapted (Carthey and Blumstein 2018, Wallach et al. 2022). However, the available evidence shows that animals respond with similar wariness to their native and introduced predators (Banks et al. 2018, Wallach et al. 2022). Wooster and colleagues (2024) found that the behavioral profile of small mammal responses to fox scent was essentially the same outside the foxes’ native range (Australia) as inside (Israel and United States). There is also no evidence that threatened species are less responsive to introduced predators than are nonthreatened species (Banks et al. 2018) nor that prey are less responsive to predators introduced most recently (Wallach et al. 2022). Second, attributing predators with extinction requires evidence that generalist predators such as foxes and cats continue hunting prey that are becoming increasingly rare and therefore difficult to find (Hayward 2011). Although there are situations in which this could plausibly happen (Baudrot et al. 2016), switching from rare to common prey appears to be the norm (e.g., Kjellander and Nordström 2003).

Finally, some have argued that the evidence for the role of foxes and cats in extirpation should not be analyzed in isolation but, rather, should be integrated into a range of ecological variables that drive decline, such as herbivory and fire (e.g., Glen and Dickman 2005, Allen 2011, Fisher et al. 2014, Doherty et al. 2015b). Indeed, we have focused our own research into integrating the claim about the effects of foxes and cats into a broader ecological hypothesis: trophic cascades. We and others have argued that the suppression and local eradication of Australia's apex predator, the dingo (*Canis dingo*), from much of the continent caused mesopredator release, which, in turn, drove extinctions (Wallach et al. 2009, 2010, Ripple et al. 2014). Although these propositions might be valid (and, indeed, we do still find them persuasive), as long as the foundational part of the hypothesis remains uncertain (introduced predators drive extinctions), larger, more complex hypotheses must be regarded with even more uncertainty (e.g., introduced mesopredators drive extinctions because apex predators are suppressed). It is also noteworthy that there are competing hypotheses, including disease, land clearing, and herbivory (e.g., Abbott 2006, Cooke 2013, Reside et al. 2017). These hypotheses should all remain on the table as localized or possibly broad explanations.

Sweeping claims have been made about introduced predators with ambiguous, weak, and—in most cases—no evidence. This challenges the notion that a core paradigm of conservation biology is evidence based, at least in Australia. It should be widely agreed that a claim should not be accepted on the basis of expert opinion (Lambert et al. 2020). For science to be of use to the process of deliberation and understanding in society, professionals ought to be wary of solidifying narratives around expert opinion and to be willing to be transparent about what is known and what remains uncertain. Ultimately, the honest answer to the question of what drove the extinction of some of Australia's small mammals is that we do not know.

## How the review was conducted

### Attributed species

We compiled a list of mammals within Australia and its nearby islands (excluding Christmas Island) that are categorized as threatened (including near threatened, vulnerable, endangered, and critically endangered; *n* = 112 species) and extinct (*n* = 25) by the IUCN Red List (IUCN 2024). We then recorded which of these mammals’ decline has been attributed to foxes or cats. We determined attribution according to claims made on the Red List species’ page “Threat” tab (IUCN 2024), and according to the list of threatened mammals attributed to foxes and cats in the Australian government's Environment Protection and Biodiversity Conservation Act 1999 (EPBC) Threat Abatement Plans (DEWHA 2013, DCCEEW 2015). The threatened status of 64 extant mammals and the extinction of 18 mammals were attributed to these predators. The IUCN and EPBC attribution of threat to foxes and cats differed in the case of 19 threatened mammals (11 attributed only by the Red List and 8 only by the EPBC). We included all species attributed by either source.

### Timeline analysis

We reviewed the literature for extirpation records of threatened and extinct mammals. For the extinct mammals, we relied on the Red List for information on the place and time they were last recorded (IUCN 2024). For the extant mammals, we searched Google Scholar with the combined terms of the threatened mammal's Latin name (e.g., *Macrotis lagotis*), *extinction*, and the name of each state and territory (e.g., *Queensland*). For the mammals now restricted to islands, we also included the term *mainland*. We viewed the articles that appeared on the first page of hits (the first 10 articles) for each mammal and snowball searched the reference lists of these articles for additional sources. We searched the text for the last records of extinct and threatened mammals, and we also included extirpated populations of least concern mammals if they were noted. Each last record was defined as confirmed (e.g., remains) or unconfirmed (e.g., sighting) by the source. We used only confirmed records in our analyses, and where there was disagreement in the literature on the last dates, we included both. We used the arrival timelines from the distribution maps produced by Fairfax (2019) for foxes and by Abbott (2008) for cats, which we digitized using QGIS v3.10. We adapted the distribution map by Fairfax (2019) to exclude foxes from the tropics (following the post-1930 contour line) and from Tasmania, because there are no confirmed records of foxes in either region (Saunders et al. 1995, Marks et al. 2017). We further confirmed whether these predators established on Australia's offshore islands where mammals were extirpated (following DCCEEW 2016).

We compared the last record date and place of the threatened and extinct mammals with the first record of foxes and cats at the same place by extracting fox and cat arrival times from digitized range expansion maps with the function “extract” in the R package terra (Hijmans 2023). We added the data compiled by Abbott (2001), who plotted a set of historic local records of the last bilby sightings (*Macrotis lagotis*) relative to the first fox sightings, which we downloaded using ImageJ (where we could not distinguish between plot points, we took a center point). The full data set is presented in table S1.

Timeline records contain documented uncertainty in both time and space. Uncertainty in extirpation time was based on the accuracy of record dates (e.g., the 1960s included 1960–1969). The uncertainty in predator arrival times was 5 years per region for foxes, following Fairfax (2019), and 10 years per region for cats, following Abbott (2008). Further uncertainty was accounted for according to spatial specificity of extirpation records. Uncertainty in extirpation location was based on the specificity of the record. More precise locales (e.g., towns) were overlayed with a 10 kilometer radius with the predator arrival region. For records with imprecise locales (e.g., states), we downloaded or created polygons of the region. We then overlayed these extirpation areas with the full range of possible predator arrival times provided by Fairfax (2019) and Abbott (2008). The exceptions were the timeline data for bilbies and foxes downloaded from Abbott (2001), reporting precise local record comparisons where no uncertainty ranges were provided.

Some species had several extirpation records from different locales. To give each species a similar weight, we summarized the proportion of populations with last records before and after predator arrival and then averaged the proportions across all species. We mapped the locations of extirpation and arrival records. Using Moran's *I*, we tested whether there was nonrandom spatial clustering among extirpation records that precede predator arrival records.

We summarized the proportion of populations, and the proportion of populations per species, with records before predator arrival, and those with record ranges that span before and after predator arrival dates. We considered both these records as being not in support of the hypothesis. Note we do not claim that these records *negate* the hypothesis. Records rarely fully account for history. The range of extirpation records arises from patchy surveys, and the predator arrival maps produced by Fairfax (2019) and Abbott (2008), which are based on the limited accounts available to them, are patchy as well. In most cases, the timeline data cannot be used to determine that extirpations definitely occurred before predator arrival, but it can be used to review the state of evidence available at present to support the hypothesis that these predators caused extirpations.

### Systematic review

We reviewed the literature with the search terms: the threatened species’ Latin name (e.g., *Macrotis lagotis*) or common name (e.g., *bilby*) with *Vulpes vulpes, Felis catus, red fox, cat*, and *feral cat*, in the search engines Google Scholar, Web of Science, and Scopus (supplemental figure S3). We also identified seven reviews (Woinarski et al. 2014, Doherty et al. 2015a, 2017, Moseby et al. 2015, Woolley et al. 2019, Stobo-Wilson et al. 2021, Tulloch et al. 2022) and searched their databases and reference lists for additional sources. We included all relevant published articles in these lists unless we were unable to locate the source. Finally, we searched the articles cited in the Red List pages and in Australian government threatened species recovery plans, where these were available. We included peer-reviewed articles, government reports, and PhD theses and all observations, reports, data sets, and analyses. Where identical information was reported in several articles, we selected the peer-reviewed and most updated source.

We did not exclude any piece of evidence suggesting that foxes and cats contribute to threatened mammal decline, regardless of quality. However, we did exclude accounts of scavenging or predation that were based solely on personal communications of observations lacking any further detail (e.g., location), and we also excluded predation accounts of pet cats. Our goal was to collate every possible verifiable account of a link between threatened mammals and foxes and wild cats, so we allowed for the inclusion of additional sources after the end of the formal review and until project completion. Despite this effort, some articles would have undoubtedly been missed, and we did fail to locate several sources that were referenced in the reviews but were unavailable online or in our institutional libraries.

Our initial search identified 591 articles that reported on the ecology and behavior of the threatened mammals that also mentioned foxes and cats somewhere in the text. We read through all these entries and included in our database any article that reports primary evidence (i.e., original data or observations) associating foxes and cats to the threatened mammal, including nonexperimental and observational descriptions. Structured data collection ended in March 2023 with 151 articles (figure S3). After this time, additional articles were found through reviewer recommendations and unstructured searches. Our final database included 163 articles that report associations between threatened mammals and foxes (*n* = 107 articles) and cats (*n* = 111; table S2).

For each article, we recorded the threatened mammal and predator studied, study site, type of evidence, and whether the article findings are in support of the hypothesis that foxes and cats drive declines (table 2). Where statistical analyses were presented, we used the results to assign support. Where statistical analyses were not presented, we used the author's description and interpretation to assign support. If only raw data was provided, we assigned support on the basis of whether the correlation was negative. Some articles included several observations, experiments, and species, and we included each as distinct studies.

We identified 11 types of studies, which we grouped into three main categories (predation, baiting, and population) and categorized them as being in support or not in support of the hypothesis that foxes and cats drive threatened mammal declines, on the basis of the study descriptions and statistics (table 2). Predation included scavenging, hunting, and dietary preference studies. Baiting studies were those associating poison baiting treatments directly to threatened mammal abundance without reporting on the abundance of the targeted predator. We found no studies associating other killing methods (e.g., shooting) with threatened mammal abundance that did not provide information on the predator (and was therefore included in population studies). We categorized all studies reporting associations between predator abundance and threatened mammal abundance as population studies (studies that used poison baiting but reported predator abundance were included).

To determine which of the studies were designed to test predator–prey interactions, we recorded whether predator and prey abundance estimates were provided (including abundance, activity, or occupancy). For studies that provided both, we recorded whether the sample size was greater than 1, the study had a control or spatial variation, and whether at least one other variable, confounder, or competing hypothesis was accounted for. We highlighted which species had studies that met all these qualities. We also checked whether any study followed a full experimental design (e.g., before–after control–impact).

Foxes and cats were typically listed among several causes of extirpation. We used a standardized threat rating provided by the Australian government's threat abatement plans, a qualitative, expert opinion based, assessment of fox and cat risk to each threatened mammal (DEWHA 2013, DCCEEW 2015; the Red List does not provide a standardized threat rating). The cat plan's threat rating categorizes both predators as minor, moderate, high, very high, or extreme threats (and intermediate threats; e.g., high to very high), on the basis of expert opinion offered in The Action Plan for Australian Mammals 2012 (Woinarski et al. 2014) and the Australian government's Species Profile and Threats Database (DCCEEW 2015). The fox plan's degree of threat, categorizes foxes as secondary, a threat, major, or unknown on the basis of expert opinion offered in the threatened species recovery plans and listings (DEWHA 2013). We chose to use the categories in the cat plan because it also rated foxes if they were considered an equal or higher threat than cats (Woinarski et al. 2014). Seven species were rated only in the fox plan. We assigned them one threat level lower than the rating assigned to cats unless they were categorized a major threat, in which case we assigned them an equal threat rating to cats. We summarized for each threatened mammal, in each threat rating, the number of studies for each evidence category and type, in support and not in support of the hypothesis (table 2).

### Meta-analysis

We sourced data sets for the meta-analysis from the population studies with data found in the systematic review. The only requirement for inclusion in the meta-analysis was that the study provided data for both predator and prey. All experimental methods were permitted (e.g., poison baiting treatments, exclusion fence comparisons, natural experiment). We did not include studies categorized as baiting, because those did not provide data on predator abundance. Poison baiting does not necessarily reduce fox or cat abundance (e.g., Wallach et al. 2010), and baits are often taken by species other than the intended predators (e.g., Fairbridge et al. 2003, Bengsen 2014, Moseby et al. 2021). We included all studies that provided raw predator and prey population data from which we could calculate correlation coefficients. We digitized plotted data using ImageJ. The final data set included 19 articles from which we were able to extract 45 predator correlations with 14 threatened mammals (supplemental tables S3 and S4).

We calculated Fisher's *r* to *z* transformed correlation coefficients as effect sizes to assess the relationship between predator and threatened mammal abundances. We used the number of data points as the sample size. Fisher's correlation coefficients are a commonly used effect size of the relationship between two continuous variables and they were calculated in the R package metafor v.3.9-10 with the function “escalc” and measure “ZCOR” (Viechtbauer 2010). Mixed effect meta-analytic models were fit with the function rma.mv (package metafor), while treating observation ID nested within study ID as random effect. Each correlation coefficient was weighted by its sampling variance (determined by sample size and calculated by “escalc”).

We also ran each model with either phylogenetic covariance matrix or a nonphylogenetic species ID tag to control for possible confounding influences of phylogeny or species identity. Doing so did not improve model quality (measured with BIC) in any comparison and led to minimal shifts in overall model estimates and significance (delta intercept term = 0 to –0.02) and did not account for any unexplained heterogeneity (average *I*^2^ phylogeny = 0%, species ID = 0). Finally, we reported whether the species included in the meta-analysis represent a random sample of the attributed species in terms of body mass.

## Supplementary Material

biaf046_Supplemental_Files

## Author Biography

Arian Wallach (a.wallach@qut.edu.au) is affiliated with the School of Biology and Environmental Science, in the Faculty of Science at Queensland University of Technology, in Brisbane, Queensland, Australia. Erick Lundgren (erick.lundgren@gmail.com) is affiliated with the Center for Open Science and Synthesis in Ecology and Evolution, in the Department of Biology at the University of Alberta, in Edmonton, Alberta, Canada.
