# Supplementary material for: Review of evidence that foxes and cats cause extinctions of Australia's endemic mammals
Source: Bioscience. 2025 Apr 10;75(8):615–27. doi: 10.1093/biosci/biaf046 (PMC12352315; doi:10.1093/biosci/biaf046)
Supplement: biaf046_Supplemental_Files [file biaf046_supplemental_files.zip › Figure S1-cats before after and by site copy.pdf]

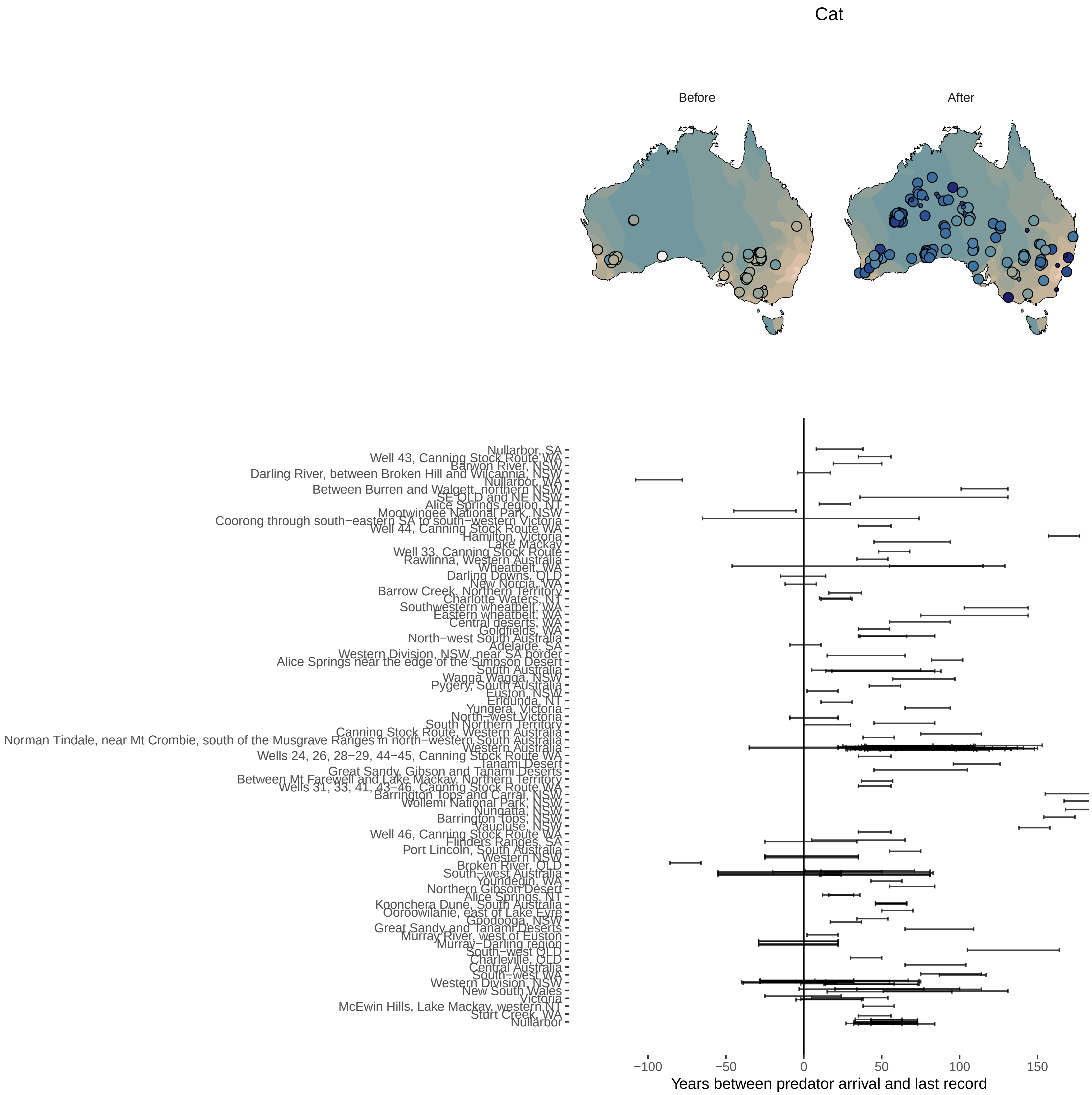
Cat
Before
After
Nullarbor, SA
Well 43, Canning Stock Route WA
Barwon River, NSW
Darling River, between Broken Hill and Wilcannia, NSW
Nullarbor, WA
Between Burren and Walgett, northern NSW
SE QLD and NE NSW
Alice Springs region, NT
Mootwingee National Park, NSW
Coorong through south−eastern SA to south−western Victoria
Well 44, Canning Stock Route WA
Hamilton, Victoria
Lake Mackay
Well 33, Canning Stock Route
Rawlinna, Western Australia
Wheatbelt, WA
Darling Downs, QLD
New Norcia, WA
Barrow Creek, Northern Territory
Charlotte Waters, NT
Southwestern wheatbelt, WA
Eastern wheatbelt, WA
Central deserts, WA
Goldfields, WA
North−west South Australia
Adelaide, SA
Western Division, NSW, near SA border
Alice Springs near the edge of the Simpson Desert
South Australia
Wagga Wagga, NSW
Pygery, South Australia
Euston, NSW
Eridunda, NT
Yungera, Victoria
North−west Victoria
South Northern Territory
Canning Stock Route, Western Australia
Norman Tindale, near Mt Crombie, south of the Musgrave Ranges in north−western South Australia
Western Australia
Wells 24, 26, 28−29, 44−45, Canning Stock Route WA
Tanami Desert
Great Sandy, Gibson and Tanami Deserts
Between Mt Farewell and Lake Mackay, Northern Territory
Wells 31, 33, 41, 43−46, Canning Stock Route WA
Barrington Tops and Carral, NSW
Wollemi National Park, NSW
Nungatta, NSW
Barrington Tops, NSW
Vaucluse, NSW
Well 46, Canning Stock Route WA
Flinders Ranges, SA
Port Lincoln, South Australia
Western NSW
Broken River, QLD
South−west Australia
Youndegin, WA
Northern Gibson Desert
Alice Springs, NT
Koonchera Dune, South Australia
Ooroowilanie, east of Lake Eyre
Goodooga, NSW
Great Sandy and Tanami Deserts
Murray River, west of Euston
Murray−Darling region
South−west QLD
Charleville, QLD
Central Australia
South−west WA
Western Division, NSW
New South Wales
Victoria
McEwin Hills, Lake Mackay, western NT
Sturt Creek, WA
Nullarbor
−100
−50
0
50
100
150
Years between predator arrival and last record
