# Supplementary material for: Review of evidence that foxes and cats cause extinctions of Australia's endemic mammals
Source: Bioscience. 2025 Apr 10;75(8):615–27. doi: 10.1093/biosci/biaf046 (PMC12352315; doi:10.1093/biosci/biaf046)
Supplement: biaf046_Supplemental_Files [file biaf046_supplemental_files.zip › Figure S1-foxes before after and by site copy.pdf]

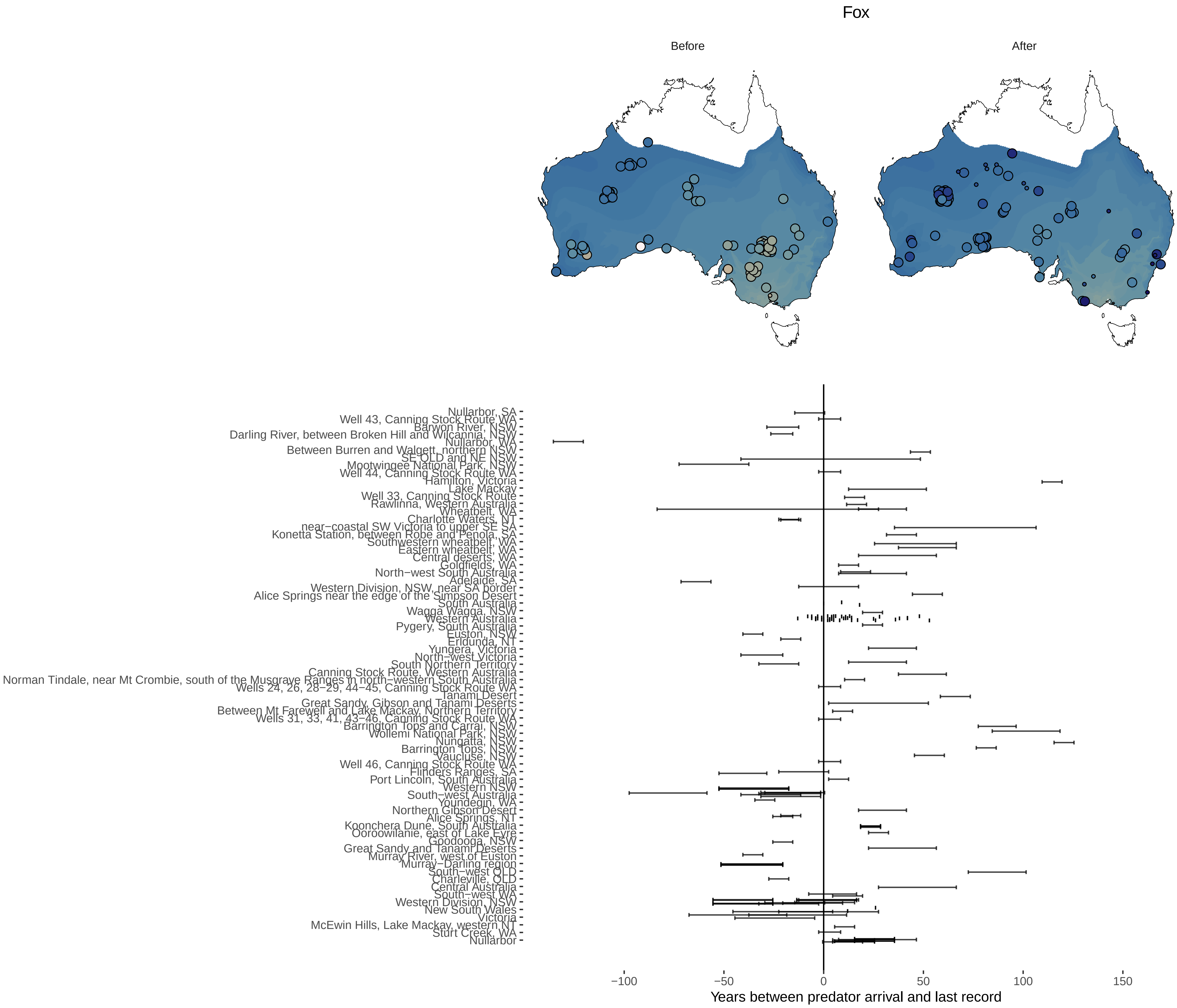
Fox
Before
After
Nullarbor, SA
Well 43, Canning Stock Route WA
Barwon River, NSW
Darling River, between Broken Hill and Wilcannia, NSW
Nullarbor, WA
Between Burren and Walgett, northern NSW
SE QLD and NE NSW
Mootwingee National Park, NSW
Well 44, Canning Stock Route WA
Hamilton, Victoria
Lake Mackay
Well 33, Canning Stock Route
Rawlinna, Western Australia
Wheatbelt, WA
Charlotte Waters, NT
near−coastal SW Victoria to upper SE SA
Konetta Station, between Robe and Penola, SA
Southwestern wheatbelt, WA
Eastern wheatbelt, WA
Central deserts, WA
Goldfields, WA
North−west South Australia
Adelaide, SA
Western Division, NSW, near SA border
Alice Springs near the edge of the Simpson Desert
South Australia
Wagga Wagga, NSW
Western Australia
Pygery, South Australia
Euston, NSW
Erldunda, NT
Yungera, Victoria
North−west Victoria
South Northern Territory
Canning Stock Route, Western Australia
Norman Tindale, near Mt Crombie, south of the Musgrave Ranges in north−western South Australia
Wells 24, 26, 28−29, 44−45, Canning Stock Route WA
Tanami Desert
Great Sandy, Gibson and Tanami Deserts
Between Mt Farewell and Lake Mackay, Northern Territory
Wells 31, 33, 41, 43−46, Canning Stock Route WA
Barrington Tops and Carrai, NSW
Wollemi National Park, NSW
Nungatta, NSW
Barrington Tops, NSW
Vaucluse, NSW
Well 46, Canning Stock Route WA
Flinders Ranges, SA
Port Lincoln, South Australia
Western NSW
South−west Australia
Youndegin, WA
Northern Gibson Desert
Alice Springs, NT
Koonchera Dune, South Australia
Ooroowilanie, east of Lake Eyre
Goodooga, NSW
Great Sandy and Tanami Deserts
Murray River, west of Euston
Murray−Darling region
South−west QLD
Charleville, QLD
Central Australia
South−west WA
Western Division, NSW
New South Wales
Victoria
McEwin Hills, Lake Mackay, western NT
Sturt Creek, WA
Nullarbor
−100
−50
0
50
100
150
Years between predator arrival and last record
