# Supplementary figures and images for: Review of evidence that foxes and cats cause extinctions of Australia's endemic mammals
Source: Bioscience. 2025 Apr 10;75(8):615–27. doi: 10.1093/biosci/biaf046 (PMC12352315; doi:10.1093/biosci/biaf046)

Fox

Cat

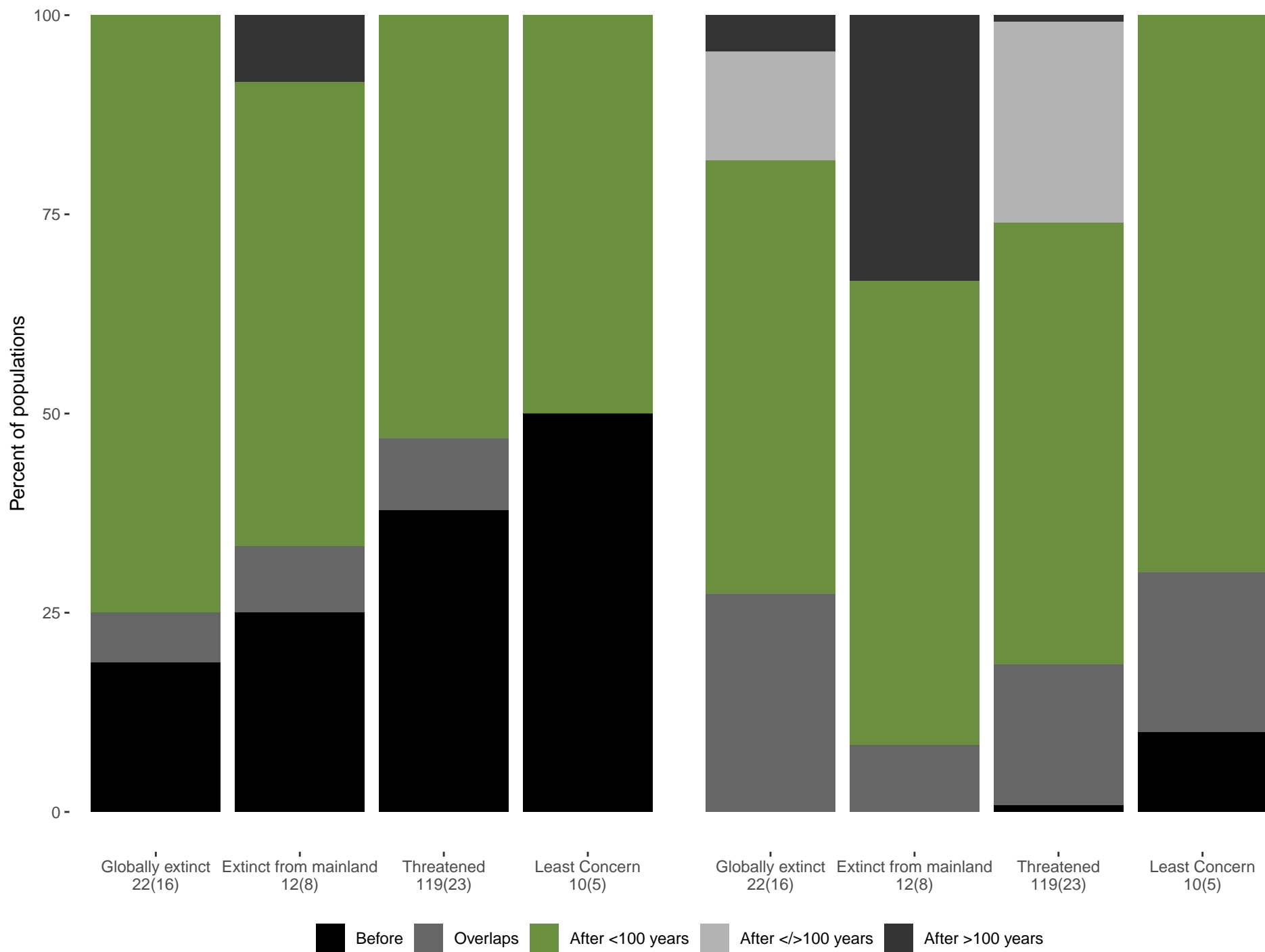

Supplement: biaf046_Supplemental_Files [file biaf046_supplemental_files.zip › Figure S2.pdf]

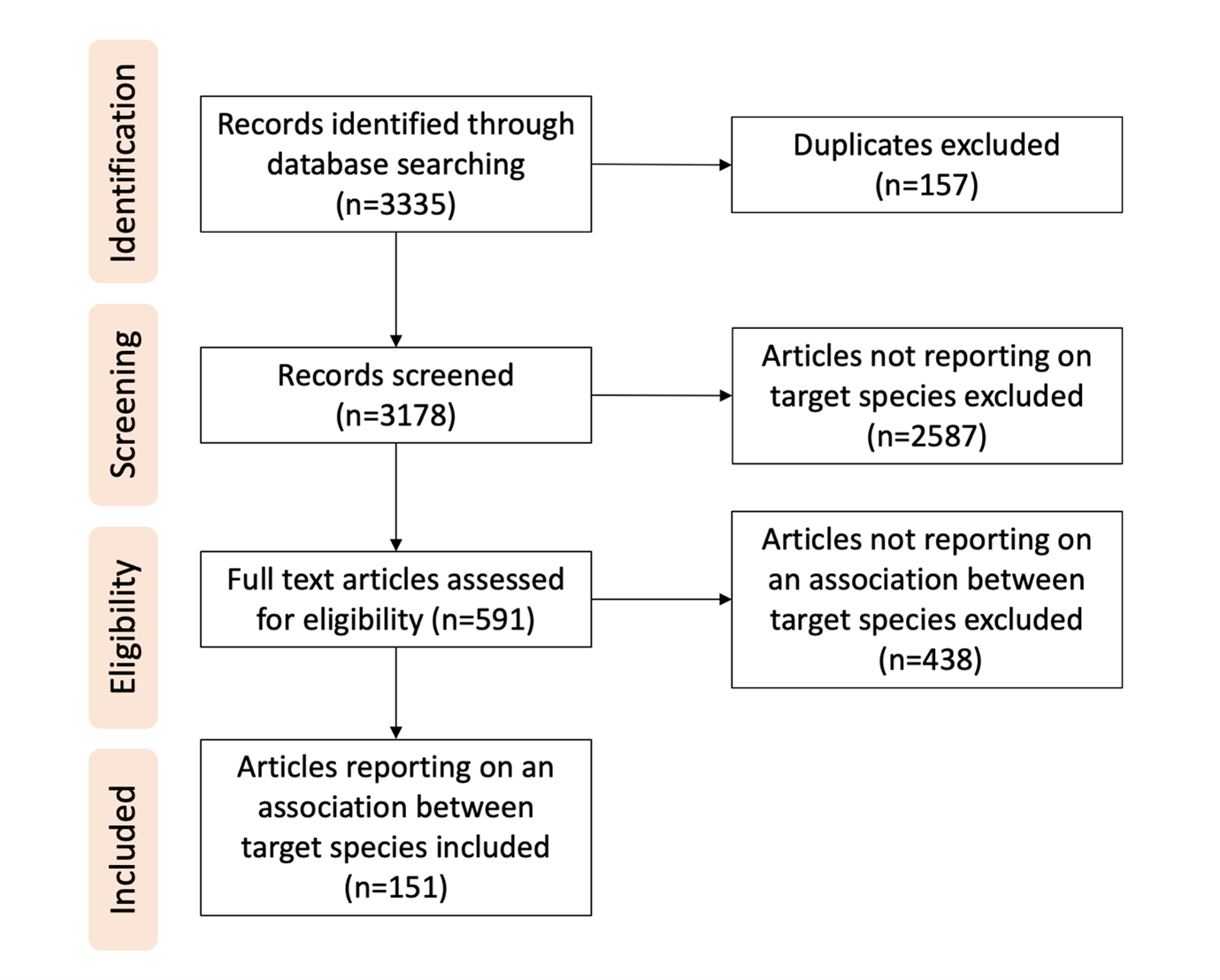

Supplement: biaf046_Supplemental_Files [file biaf046_supplemental_files.zip › Figure S3.png]

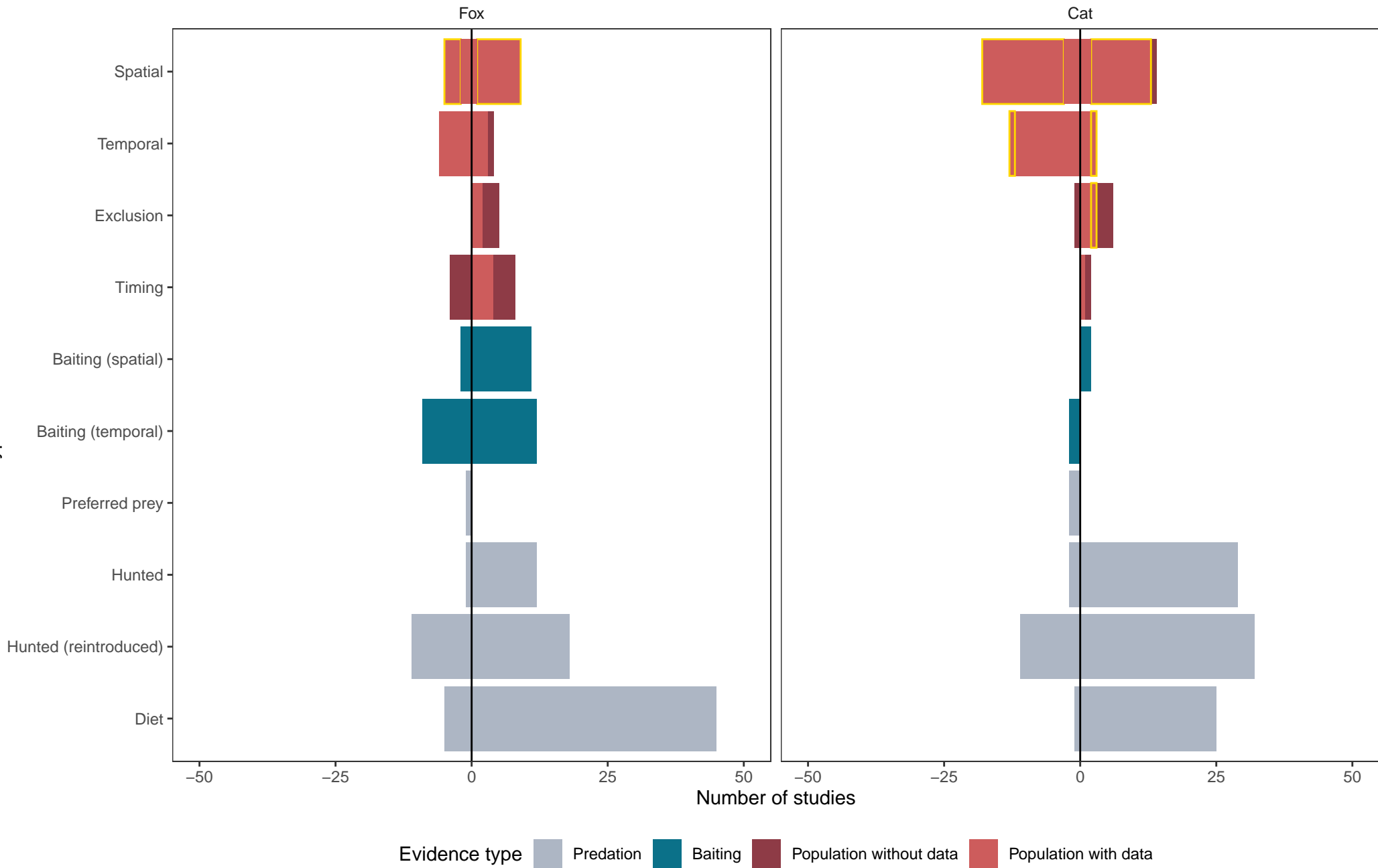

Supplement: biaf046_Supplemental_Files [file biaf046_supplemental_files.zip › Figure S4.pdf]

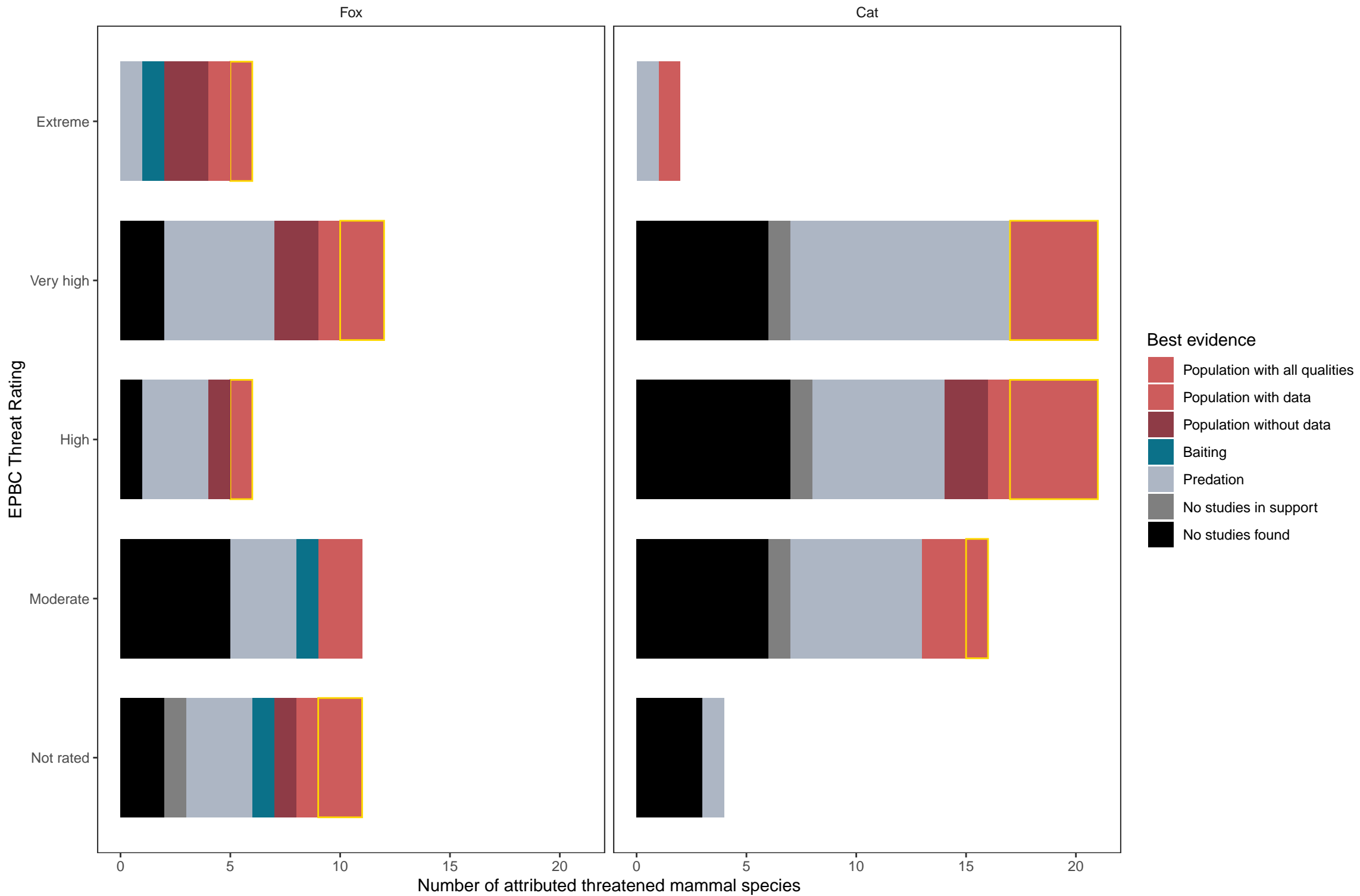

Supplement: biaf046_Supplemental_Files [file biaf046_supplemental_files.zip › Figure S5.pdf]

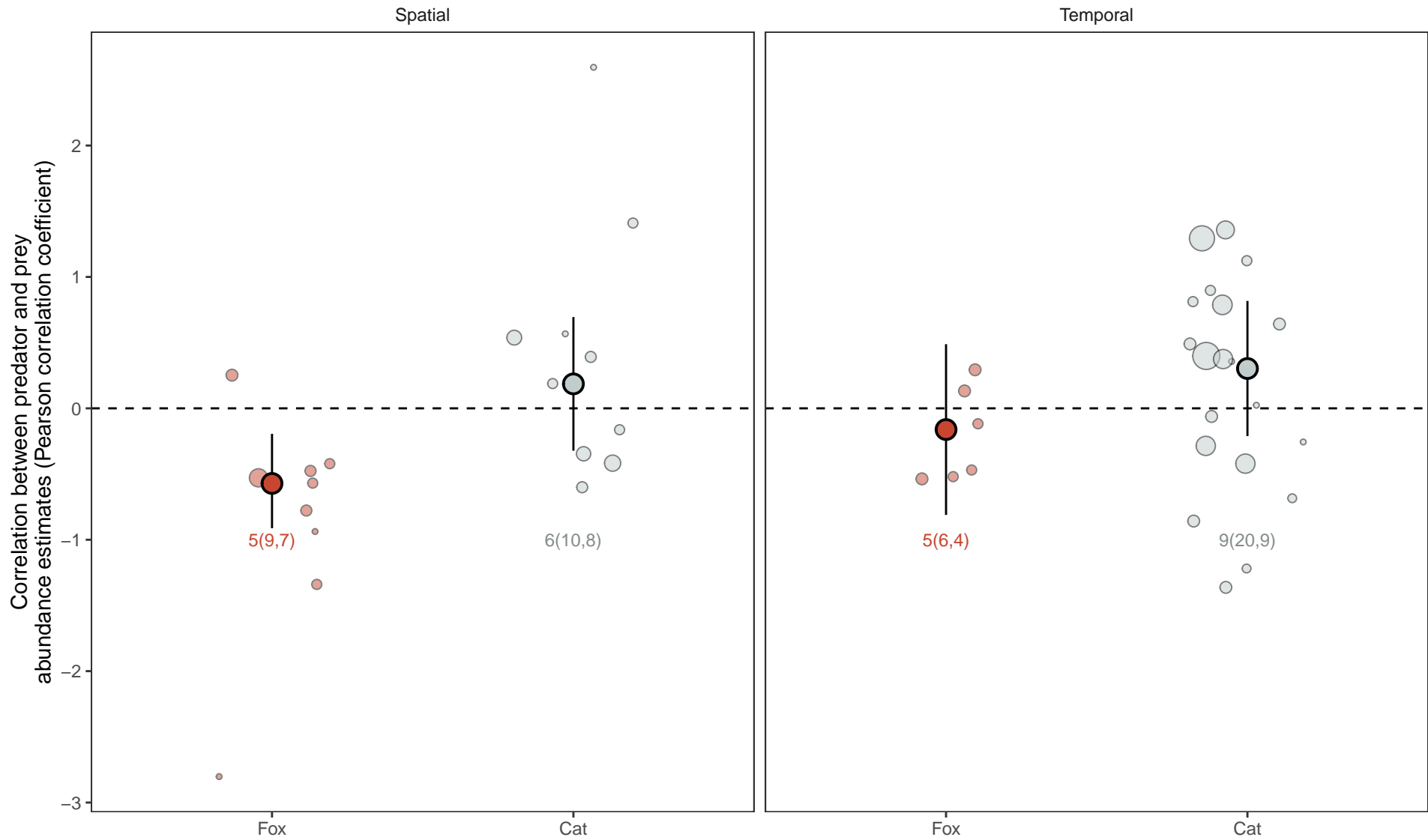

Supplement: biaf046_Supplemental_Files [file biaf046_supplemental_files.zip › Figure S6.pdf]
